# Granger causality vs. dynamic Bayesian network inference: a comparative study

**DOI:** 10.1186/1471-2105-10-122

**Published:** 2009-04-24

**Authors:** Cunlu Zou, Jianfeng Feng

**Affiliations:** 1 Department of Computer Science, University of Warwick, Coventry, UK; 2 Centre for Computational Systems Biology Fudan University, Shanghai, PR China

## Abstract

**Background:**

In computational biology, one often faces the problem of deriving the causal relationship among different elements such as genes, proteins, metabolites, neurons and so on, based upon multi-dimensional temporal data. Currently, there are two common approaches used to explore the network structure among elements. One is the Granger causality approach, and the other is the dynamic Bayesian network inference approach. Both have at least a few thousand publications reported in the literature. A key issue is to choose which approach is used to tackle the data, in particular when they give rise to contradictory results.

**Results:**

In this paper, we provide an answer by focusing on a systematic and computationally intensive comparison between the two approaches on both synthesized and experimental data. For synthesized data, a critical point of the data length is found: the dynamic Bayesian network outperforms the Granger causality approach when the data length is short, and vice versa. We then test our results in experimental data of short length which is a common scenario in current biological experiments: it is again confirmed that the dynamic Bayesian network works better.

**Conclusion:**

When the data size is short, the dynamic Bayesian network inference performs better than the Granger causality approach; otherwise the Granger causality approach is better.

## Background

Based upon high throughput data, to reliably and accurately explore the network structure of elements (genes, proteins, metabolites, neurons etc.) is one of the most important issues in computational biology [1-6]. Currently, there are two main approaches which are often used to infer causal relationships [7] or interactions among a set of elements [8,9]. One is the Granger causality approach [10,11], and the other is the Bayesian network inference approach [12,13]. The latter is often applied to static data. However, one can employ the dynamic Bayesian networks to deal with time series data for which the Granger causality has been solely developed. The Granger causality has the advantage of having a corresponding frequency domain decomposition so that one can clearly find at which frequencies two elements interact with each other.

Giving a multi-variable time series dataset, the Granger causality and dynamic Bayesian networks [14] can both be applied. The Granger causality notation, which was firstly introduced by Wiener and Granger [15,16], proposed that we can determine a causal influence of one time series on another: the prediction of one time series can be improved by incorporating the knowledge of the second one. On the other hand, The Bayesian network [17] is a special case of a diagrammatic representation of probability distributions, called probabilistic graphical models [18-20]. The Bayesian network graph model comprises nodes (also called vertices) connected by directed links (also called edges or arcs) and there is no cycle in the graph. To learn the structure and the parameters for the Bayesian networks from a set of data, we should search the space(s) of all possible graph representations, and find out which structure is most likely to produce our data. If we have a scoring function (or likelihood function) which can determine the structure and parameter likelihood from the data, then the problem is to find the highest score (maximum likelihood) structure among all the possible representations.

The causal relationship derived from these two approaches could be different, in particular when we face the data obtained from experiments. Therefore it is of vital importance to compare these two causal inferring approaches before we could confidently apply them to biological data. By doing the comparison, one expects to find the advantages, performances and stabilities for each technique.

Adopting the most common existing methods to find the coefficients of the time series in both approaches in the literature, we compare the dynamic Bayesian network with the Granger causality both in the linear and nonlinear model. Interestingly, a critical point of the data length is found. When the data length is shorter than the critical point, the dynamic Bayesian network approach outperforms the Granger causality approach. But when the data length is longer, the Granger causality is more reliable. The conclusion is obtained via intensive computations (more than 100 computers over a few weeks). A biological data set of gene microarray is analyzed using both approaches, which indicates that for a data set with a short sampling length the dynamic Bayesian network produces more reliable results. In summary, we would argue that the dynamic Bayesian network is more suitable for dealing with experimental data.

## Results

To illustrate and compare the differences between the dynamic Bayesian network inference and the conditional Granger causality, a simple multivariate model with fixed coefficients, which has been discussed in many papers to test the Granger causality, is tested first. We then extend our comparisons to the more general case of the model with random coefficients, which requires considerable computational resources. More than 100 networked computers are used to perform the comparisons for more than a week. Both the Granger causality and the dynamic Bayesian network are applied to nonlinear models. Finally, we test our approach on a set of microarray data recently acquired from a comparison of mock and infected Arabidopsis leaf.

### Synthesized data: linear case

**Figure 1:**
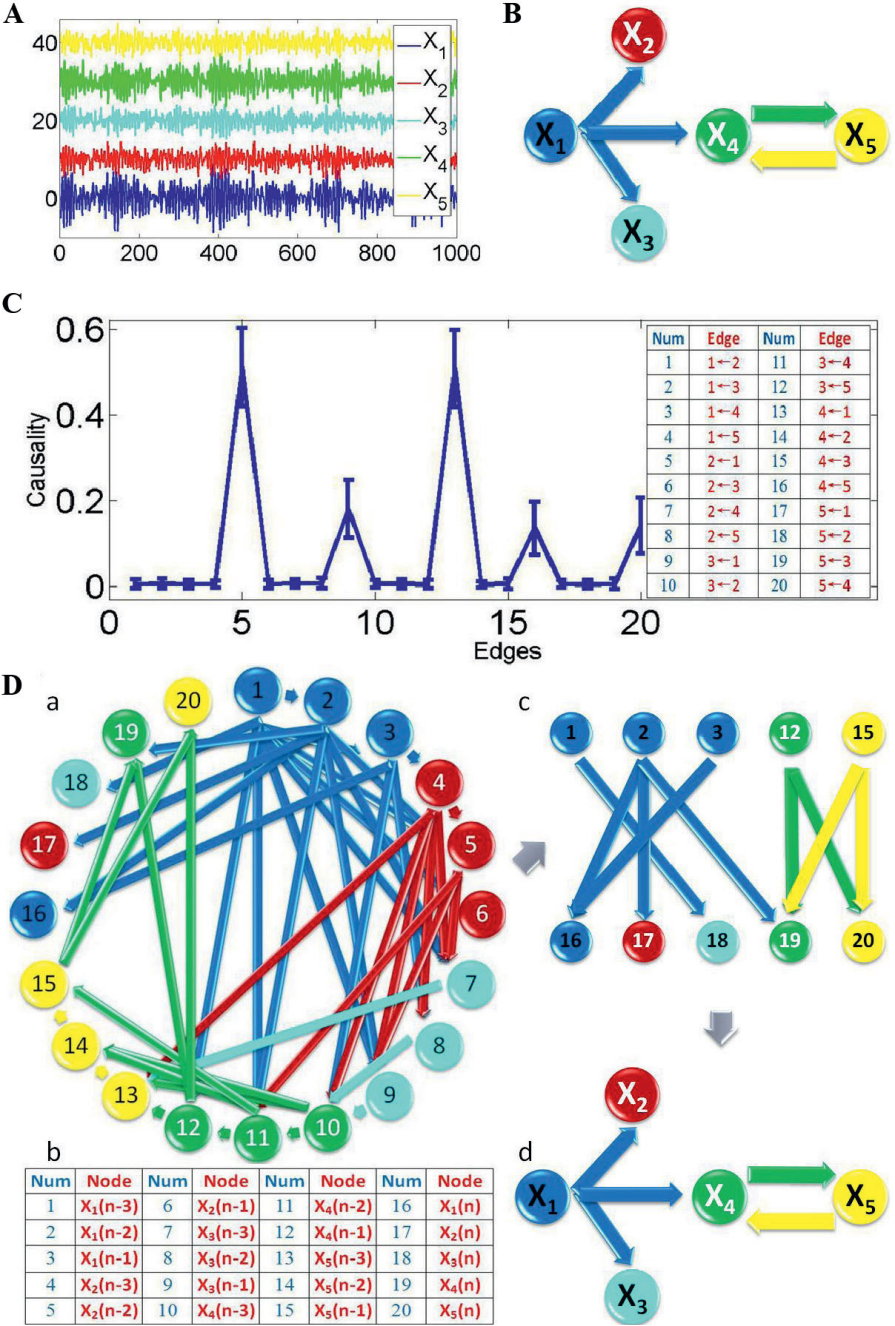
**Granger causality and Bayesian network inference approaches applied on a simple linear toy model**. A. Five time series are simultaneously generated, and the length of each time series is 1000. *X*_2_, *X*_3_, *X*_4 _and *X*_5 _are shifted upward for visualization purpose. B. Granger causality results. (a) The network structure inferred from Granger causality approach. (b) The 95% confidence intervals graph for all the possible directed connections. (c) For visualization purpose, all directed edges (causalities) are sorted and enumerated into the table. The total number of edges is 20. C. Dynamic Bayesian network inference results. (a) The causal network structure learned from Bayesian network inference. (b) Each variable is represented by four nodes, representing different time-lags, we have a total of 20 nodes. They are numbered and enumerated in the table. (c) The simplified network structure: since we only care about the causality to the current time status, we can remove all the other edges and nodes that have no connection to the node 16 to node 20 (five variables with current time status). (d). A further simplified network structure of causality.

**Example 1 **Suppose we have 5 simultaneously recorded time series generated according to the equations:

(1)

where *n *is the time, and [*ε*_1_, *ε*_2_, *ε*_3_, *ε*_4_, *ε*_5_] are independent Gaussian white noise processes with zero means and unit variances. From the equations, we see that *X*_1_(*n*) is a cause of *X*_2_(*n*), *X*_3_(*n*) and *X*_4_(*n*), and *X*_4_(*n*) and *X*_5_(*n*) share a feedback loop with each other, as depicted in Figure 1B. Figure 1A shows an example of the time trace of 5 time series. For the Granger causality approach, we simulated the fitted vector autoregressive (VAR) model to generate a data set of 100 realizations of 1000 time points, and applied the bootstrap approach to construct the 95% confidence intervals (Figure 1C). For Granger causality, we assume the causality value is Gaussian distributed. Then the confidence intervals can be obtained by calculating the mean and standard derivation values [21,22]. According to the confidence intervals, one can derive the network structure as shown in Figure 1B which correctly recovers the pattern of the connectivity in our toy model. For the dynamic Bayesian network inference approach, we can infer a network structure (Figure 1Da) for each realization of 1000 time points. The final resulting causal network model was inferred with high-confidence causal arcs (the arcs occur more than 95% of the time in the whole population) between various variables [13]. This complex network contains the information of different time-lags for each variable. It fits exactly the pattern of connectivity in our VAR model. In order to compare it with the Granger causality approach, we can further simplify the network by hiding the information of time-lags, and then we infer the exactly same structure as the Granger causality approach (Figure 1Dd). From this simple example, we can find that both approaches can reveal correct network structures for the data with a large sample size (1000 here).

**Figure 2:**
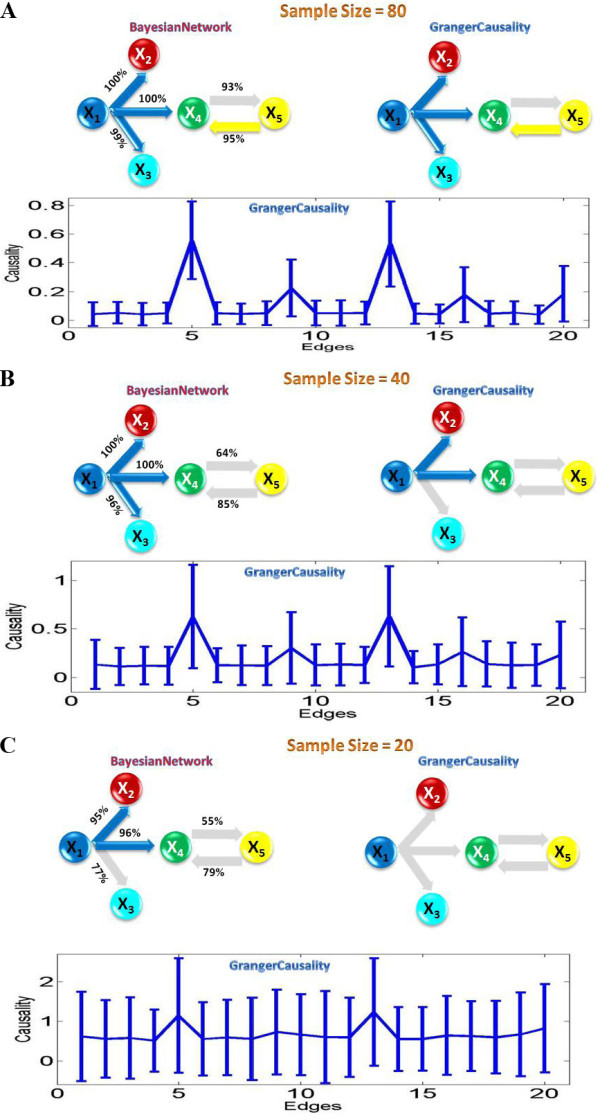
**Granger causality and Bayesian network inference applied on data points of various sample sizes**. The grey edges in the inferred network structures indicate undetected causalities in the toy model. For each sample size n, we simulated a data set of 100 realizations of n time points. The Bayesian network structure represents a model average from these 100 realizations. High-confidence arcs, appearing in at least 95% of the networks are shown. The Granger causality inferred the structure according to the 95% confidence interval constructed by using the bootstrap method. (A) The sample size is 80. (B) The sample size is 60. (C) The sample size is 20.

Most, if not all, experimental data has a very limited time step due to various experimental restrictions. Hence one of the key quantities to test the reliability of an approach is the data length (sample size). In the next setup, we reduce the sample size to a smaller value and check its impact. Figure 2A shows the case of the sample size of 80: we find both approaches start failing to detect some interactions (false negative). By reducing the sample size to 20, we can see that the Bayesian network inference can derive more true positive connections than the Granger causality. This is certainly an interesting phenomenon and we intend to explore whether it is true for a more general case.

**Example 2 **we considered a more general toy model; the coefficients in the equations (1) of Example 1 are randomly generated. This toy model aims to test the causality sensitivity for the two approaches. Suppose 5 simultaneously generated time series according to the equations:

(2)

where *W*_1_, *W*_2_, ⋯, *W*_9 _are uniformly distributed random variables in the interval [-1,1]. The randomly generated coefficients are also required to make the system stable. The stability can be tested by using the z-plane pole-zero method, which states if the outermost poles of the z-transfer function describing the time series are inside the unit circle on the z-plane pole-zero plot, then the system is stable.

The above toy model is then used to test the two different causality approaches: Bayesian network inference and Granger causality. They are applied with different sample sizes. For each sample size, we randomly generated 100 different coefficient vectors [*W*_1_, *W*_2_, ⋯, *W*_9_], which corresponds to100 different toy models in Example 1. For each different coefficient vectors model, we applied the same approach as in Example 1, using Monte Carlos method to construct 95% confidence interval for the Granger causality approach and chose high-confidence arcs (appearing in at least 95% of all samplings) for the Bayesian network inference approach. The total number of arcs (or causalities) is 500 (5 interactions for each realization) for each sample size. However we cannot expect to detect the maximum number of arcs in our system, since the coefficients are randomly generated, which could be significantly small.

**Figure 3:**
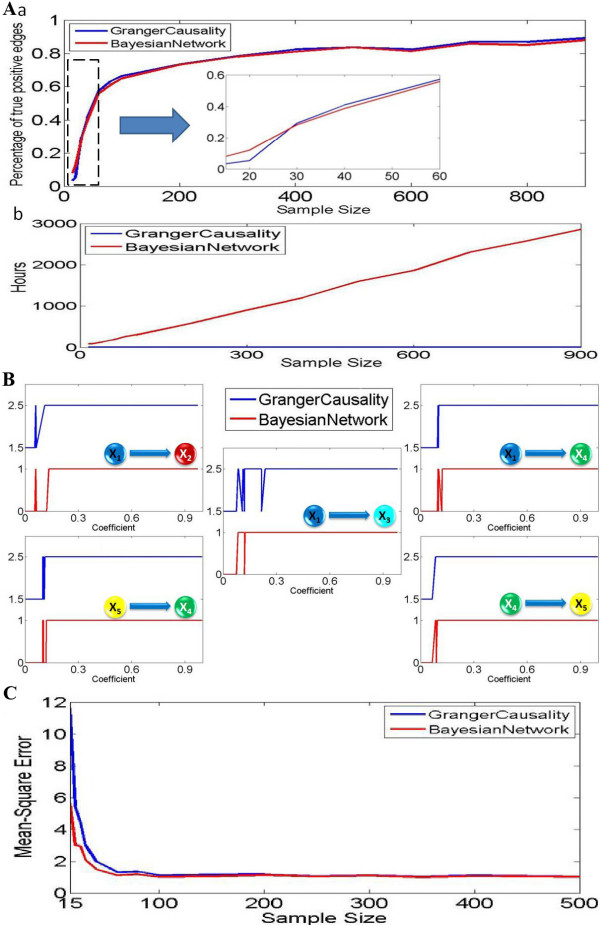
**Granger causality and Bayesian network inference applied on a stochastic coefficients toy model**. The parameters in polynomial equation are randomly generated in the interval [-1,1]. For each randomly generated coefficient vector, we applied the same approach as example 1: bootstrapping method and 95% confidence interval for Granger causality; 95% high confidence arcs are chosen from Bayesian network inference. (A) We applied both approaches on different sample size (from 20 to 900). For each sample size, we generated 100 different coefficient vectors, so the total number of directed interactions for each sample size is 500. (a) The percentage of detected true positive causalities for both approaches. (b) Time cost for both approaches. (B) For sample size 900, the derived causality (1 represents positive causality and 0 represents negative) is plotted with the absolute value of corresponding coefficients. For visualization purpose, the figure for Granger causality is shifted upward. (C) Linear model fitting comparison for both Granger causality and Bayesian networks. Using a number of training data points to fit both linear models, one can calculate a corresponding predicted mean-square error by applying a set of test data. And we can find that Bayesian networks inference approach works much better than the Granger causality approach when the sample size is significant small (around 100). When the sample size is significant large, both approaches converge to the standard error which exactly fits the noise term in our toy model.

Figure 3Aa shows the comparison result of the percentage of true positive connections derived from these two methods. In general, the Granger causality approach can infer slightly more true positive causalities compared to the Bayesian network inference approach when the data length is long. It is interesting to see that there is a critical point at around 30 in Figure 3Aa. If the sample size is larger than 30, then the Bayesian network recovers less positive connections. However, if the sample size is smaller than 30, the Bayesian network performs better. From Figure 3Ab, we see that computing time for the Bayesian network inference is much larger than the Granger causality.

Now we are in the position to find out why the dynamic Bayesian network is better than the Granger causality when the data length is short, and vise verse. In Figure 3B, we compare the performances on different coefficients (strength of interaction) for a fixed sample size of 900 (super-critical case). The x-axis shows the absolute value of coefficients, and y shows the corresponding causality (1 indicates positive causality and 0 indicates no causality). For visualization purposes, the figure for the Granger causality is shifted upward. From the five graphs, we can see that there is no difference between these two approaches if the coefficients are significant large (strong interactions with an absolute value of coefficients being greater than 0.15): both approaches can infer the correct connections. For most cases, the Granger causality approach performs with more stability when the coefficients are larger than 0.15, and the Bayesian network inference approach shows slightly more oscillations around this point. Hence we conclude that the Granger causality is less sensitive to the small value of the connection when the data length is large (see also the nonlinear case below).

We now compare the fitting accuracy of the two approaches, as shown in Figure 3C. We use the average mean-square error as a measurement of the fitting. Not surprisingly, the dynamic Bayesian network approach considerably outperforms the simple fitting algorithm in the Granger approach [15,16], in particular when the data length is short.

In conclusion, when the data is a reasonable fit to the original model, the Granger causality works better. This is due to the fact that the Granger causality approach is more sensitive to a small value of the interactions. When the data length is short, the Bayesian approach can fit the data much more reliably and it outperforms the Granger approach.

### Synthesized data: non-linear case

In real situations, all data should be nonlinear and a linear relationship as described above is only an approximation. To address the nonlinear issue, we turn our attention to kernel models. As we all know, any nonlinear relationship can be approximated by a series of kernel functions.

**Example 3 **we modify the model in example 1 to a series of nonlinear equations as follows:

(3)

**Figure 4:**
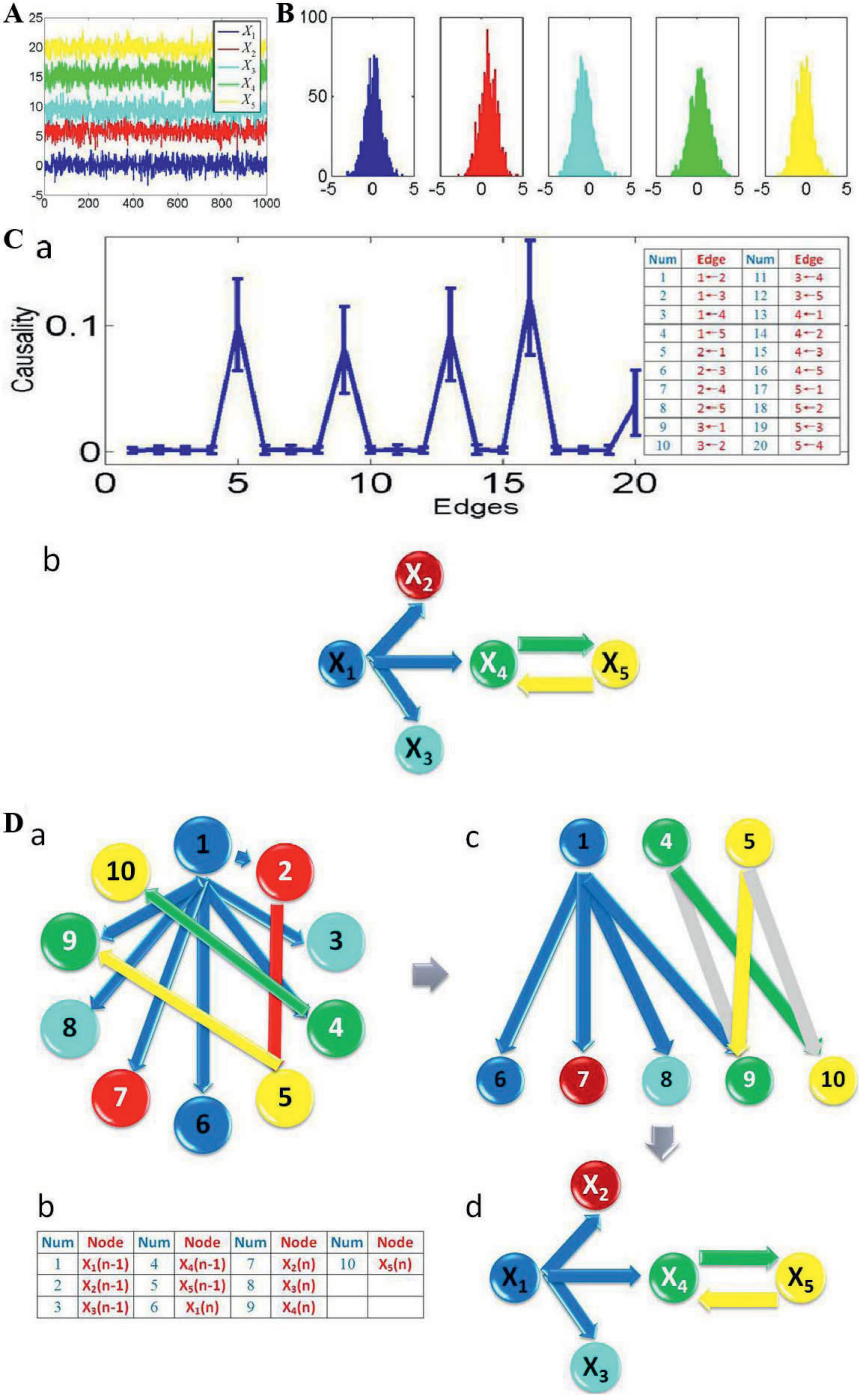
**Granger causality and Bayesian network inference approaches applied on a simple non-linear toy model**. (A) Five time series are simultaneously generated, and the length of each time series is 1000. They are assumed to be stationary. (B) The five histogram graphs show the probability distribution for these five time series. (C) Assuming no knowledge of MVAR toy model we fitted, we calculated Granger causality. Bootstrapping approach is used to construct the confidence intervals. The fitted MVAR model is simulated to generate a data set of 100 realizations of 1000 time points each. (a) For visualization purpose, all directed edges (causalities) are sorted and enumerated into the table. The total number of edges is 20. 95% confidence interval is chosen. (b) The network structure inferred from Granger causality method correctly recovers the pattern of connectivity in our MVAR toy model. (D) Assuming no knowledge of MVAR toy model we fitted, we approach Bayesian network inference. (a) The causal network structure learned from Bayesian network inference for one realization of 1000 time points. (b) Each variable is represented by two nodes; each node represents different time statuses, so we have 10 nodes in total. They are numbered and enumerated into the table. (c) The simplified network structure: since we only care about the causality to the current time status, we can remove all the other edges and nodes that have no connection to the node 6 to node 10 (five variables with current time status). (d) A further simplified network structure: in order to compare with Granger causality approach, we hid the information of time status, and we obtained the same structure as Granger causality method had.

In this example, the center and variance of each time series is chosen as the center and variance in the kernel function. We use the fuzzy c-mean method to find the center of each time series and then applied the same approach as in **Example 1**. For the Granger causality approach, we simulated the fitted VAR model to generate a data set of 100 realizations of 1000 time points, and applied the bootstrap approach to construct the 95% confidence intervals (Figure 4Ca). According to the confidence interval, one can derive the network structure (Figure 4Cb) which correctly recovers the pattern of connectivity in our non-linear model. For the Bayesian network inference approach, we can infer a network structure (Figure 4Da) for each realization of 1000 time points. We can then obtain a simplified network structure (Figure 4Dd).

**Figure 5:**
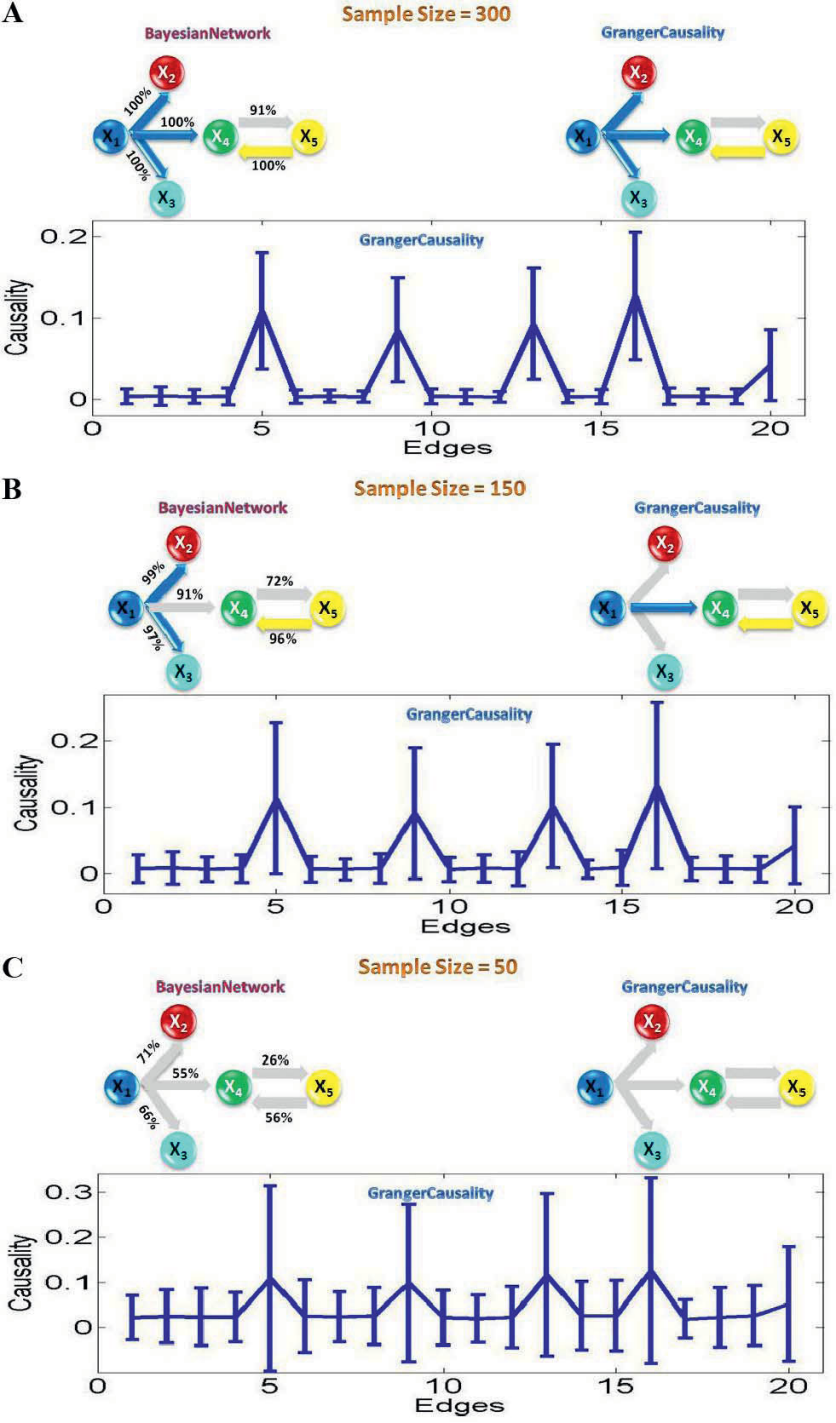
**Granger causality and Bayesian network inference applied on insufficient number of data points for non-linear model**. The grey edges in the inferred network structures indicate undetected causalities in our defined toy model. For each sample size n, we simulated a data set of 100 realizations of n time points. The Bayesian network structure represents a model average from these 100 realizations. High-confidence arcs, appearing in at least 95% of the networks are shown. The Granger causality inferred the structure according to the 95% confidence interval constructed by using the bootstrap method. (A) The sample size is 300. (B) The sample size is 150. (C) The sample size is 50.

For a small sample size (see Figure 5), worse results are obtained for both approaches comparing to the previous linear model. Both approaches start to miss interactions when the sample size is smaller than 300. When the sample size is 150, the Bayesian network inference approach can detect one more true positive interaction than the Granger causality. However, when the sample size is 50, both approaches fail to detect all the interactions.

**Figure 6:**
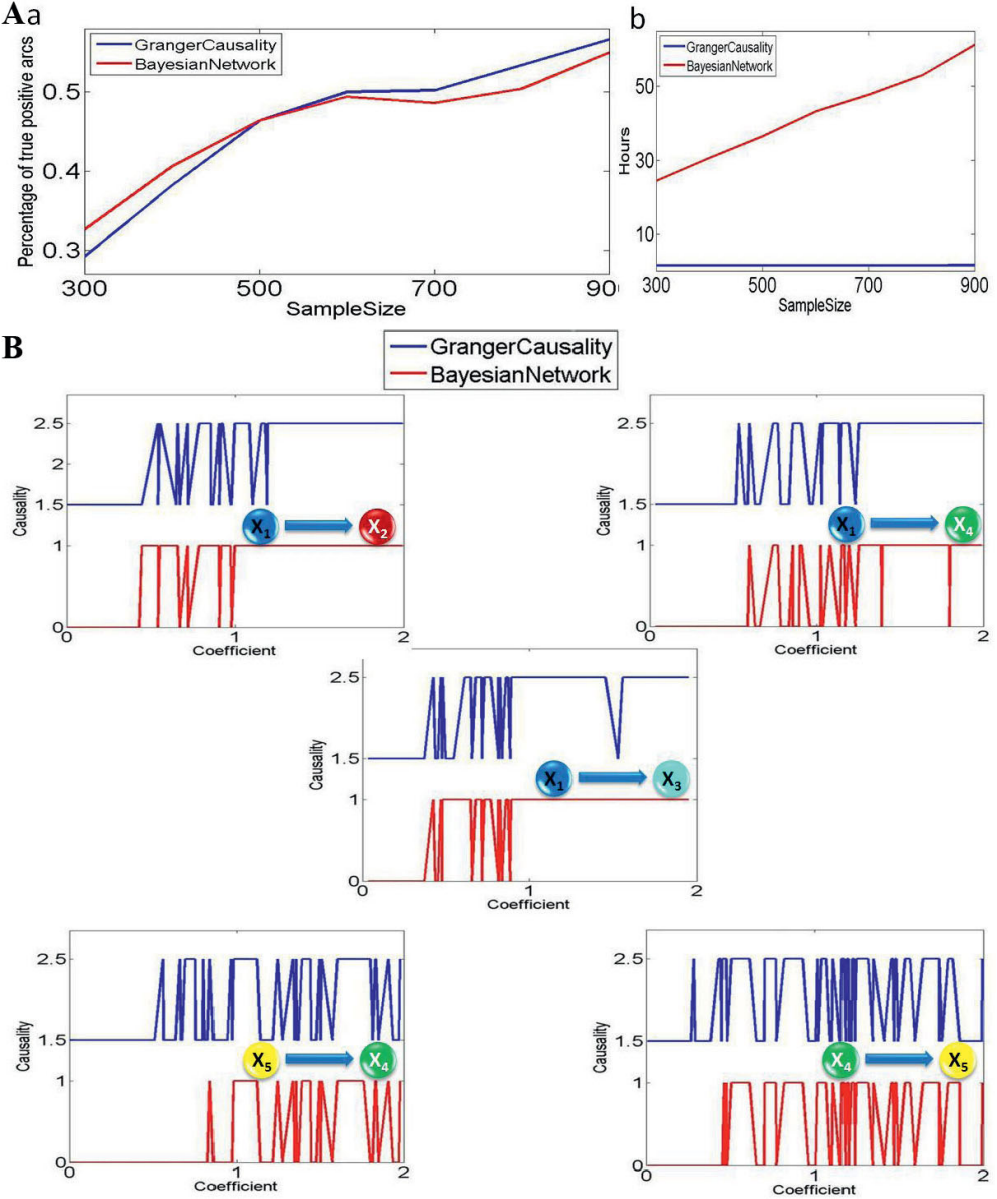
**Granger causality and Bayesian network inference applied on a stochastic coefficients non-linear model**. The parameters in polynomial equation are randomly generated in the interval [-2,2]. (A) We applied both approaches on different sample size (from 300 to 900). For each sample size, we generated 100 different coefficient vectors, so the total number of directed interactions for each sample size is 500. (a) The percentage of detected true positive causalities for both approaches. (b) Time cost for both approaches. (B) For sample size 900, the derived causality (1 represents positive causality and 0 represents negative) is plotted with the absolute value of corresponding coefficients. For visualization purpose, the figure for Granger causality is shifted upward.

In the next step, we extend our non-linear model to a more general setting in which the coefficients in the equations are randomly generated. Figure 6Aa shows the comparison result of the percentage of true positive connections derived from these two methods. It is very interesting to see that a critical point around 500 exists in the non-linear model, similar to the linear model before. From Figure 6Ab, the computing time required for the Bayesian network inference is still much larger than the Granger causality. In Figure 6B, we compare the performances on different coefficients (strength of interaction) for a fixed sample size of 900. From the five graphs, we can see that in general the Granger approach is more sensitive to a small value of the coefficients (see Figure 6B. X_5 _-> X_4 _and X_4 _-> X_5_).

Therefore, all conclusions in the linear case are confirmed in the nonlinear model. In the literature [23], the result they obtained shows the same direction as we did here, which finds that the Granger causality performs better than the dynamic Bayesian network inference concerning a nonlinear kernel model of genetic regulatory pathways and for a sufficiently large sample size (2000 data points).

### Experimental data

**Figure 7:**
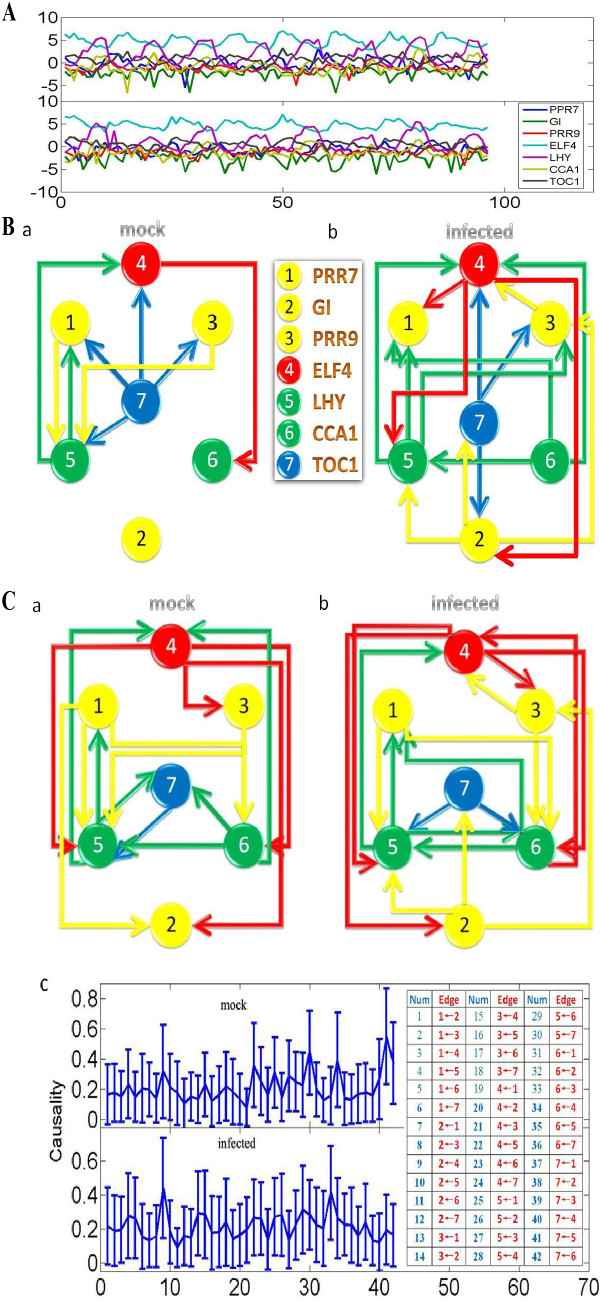
**Granger causality approaches and Bayesian network inference approaches applied on experimental data (small sample size)**. The experiment measures the intensity of 7 genes in two cases of Arabidopsis Leaf: mock (normal) and infected. (A)The time traces of 7 genes are plotted. There are 4 realizations of 24 time points. The time interval is 2 hours. (B) The network structures are derived by using dynamic Bayesian network inference. All the genes are numbered as shown. Interestingly, after infection, the total network structure is changed. (a) The network structure for mock case. (b) the network structure for infected case. (C) The network structures are derived by using Granger causality. (a) The network structure for mock case. (b) the network structure for infected case. (c) Using bootstrapping method to construct a 95% confidence intervals. For visualization purpose, all the directed edges are numbered and enumerate them into the table.

Finally we carry out a study on experimental data of microarray experiments. The gene data were collected from two cases of Arabidopsis Leaf: the mock (normal) case and the infected case with the plant pathogen *Botrytis cinerea*. A total of 31,000 genes were measured with a time interval of two hours, with a total of 24 sampling points (two days) and four replicates. We test the Granger causality approach and dynamic Bayesian network inference approach on a well-known circadian circuit. This circuit contains 7 genes: PRR7, GI, PRR9, ELF4, LHY, CCA1 and TOC1. Figure 7A shows the time traces of the 7 genes. From the time traces figure, it is clearly to see that they exhibit a 24 hour rhythm. Note that the total number of time points is only 24. Compared to our previous toy model case, this sample size is quite small. We therefore expect the Bayesian network inference to be more reliable.

We first apply the dynamic Bayesian network inference approach on these two data sets. The two network structures for two cases are shown in Figure 7B. In the next step, the conditional Granger causality approach is applied. By using the bootstrapping method, we construct 95% confidence intervals as shown in Figure 7Cc. Finally, we can also obtain two network structures for two different cases shown in Figure 7Ca and Figure 7Cb. It is clearly seen that the globe patterns for the mock case and the infected case are different.

From the literature, there are three well known connections among the whole structure for the mock case. (1) It is known that GI alone is independent of the remaining six genes in the circuit. There should be no connection to and from the GI node (node 2 in the Figure 7) in our derived network. From Figure 7Ba and Figure 7Ca, we find that the dynamic Bayesian network inference method clearly picks this up, but the conditional Granger causality approach fails to detect this property. The Granger causality approach derived two false positive arcs which were connected to a GI node as shown in Figure 7Ca. (2) It is known that PRR7 and LHY share a feedback loop. In other words, there should be two directed arcs connected from node 1 (PRR7) to node 5 (LHY) and from node 5 to node 1. The network structures derived from both approaches are in agreement with this known relationship. (3) It is known that ELF4 has some interactions with both LHY and CCA1. There should be some connections between node 4 (ELF4) to node 5 (LHY), and between node 4 (ELF4) and node 6 (CCA1). From our derived structures, both approaches can detect these connections, which are in agreement with the known structure in the literature [24,25].

According these three known relationships in the structure, we can find that the Bayesian network structure is in agreement with all three rules, but the network structure derived from the conditional Granger causality is not: two more false positive interactions are found. Again for a small sample size, the Bayesian network inference approach could be more reliable than the conditional Granger causality approach.

## Discussion

### A fair comparison

In our results presented here, one of the key issues which is the cause of the critical point of the sampling size between the dynamic Bayesian approach and the Granger causality lies in the fact that a batch fitting approach is used in the Granger causality approach. One might argue that we could use the sequential fitting approach as in the Bayesian network to improve the performance of the Granger causality approach. This is certainly the case. However, due to the thousand publications in both topics [26], we simply adopted the most common approaches in the dynamic Bayesian network approach and the Granger causality. Developing one of the approaches, for example the Granger causality, so that it could always outperform the other is an interesting future research topic.

### How long is long enough?

Although we have found the critical point of the two approaches, in practical applications, we have no certain idea where the critical point is. Hence, we still have to choose one of them to tackle the data. In molecular biology, we have to deal with a very limited data size; but in physiology, for example, neurophysiology, the data we record is usually very long. Hence one could argue that we use the dynamic Bayesian network in gene, protein or metabolite data, and apply the Granger causality to physiology data. The dynamic Bayesian network is more often reported in molecular biology, but the Granger causality has been very successfully applied in neurophysiological data [27] and fMRI. The result we chose to use was always chosen through experimental validation, as we did here for the plant data.

### Frequency decomposition

As we emphasized at the beginning, the advantage of the Granger causality over the dynamic Bayesian network is the frequency decomposition, which is usually informative when we deal with temporal data. For example, in neurophysiology data, we know the brain employs different frequency bands to communicate between neurons and brain areas [28,29]. We would expect a similar situation to arise in genes, proteins and metabolites, although we lack a detailed analysis due to the limited data length. To this end, we have also presented frequency decomposition results in Appendix 1 (see Additional files 1 and 2) for the dynamic Bayesian network.

### False positive

In our synthesized data, for both approaches, we did not find any false positive links in our experiments. However, there were a few false positive links found when we applied the conditional Granger causality and also partial Granger causality (data not shown, [21]) on the gene data. One might ask why this is the case; there are several different reasons. Firstly, the experimental data is not strictly stationary: it is a natural process and evolves with time. As a first approximation, we treat it as stationary. Of course, we could use ARIMA rather than ARMA model to fit the data in the Granger causality. Secondly, the seven gene network is only a small network embedded in complete and large network, so there are latent variables. Using the partial Granger causality [21] which was originally developed for eliminating latent variables, GI still has links with the other six genes. Whether the dynamic Bayesian network could do a better job in the presence of latent variables is another research topic.

### The meaning of the found motifs

Two circuits are found: one with the mock plant and one with the infected plant. The plant rewires its circadian circuit after infection. Ignoring the issue of identifying the molecular mechanisms which control circuit rewiring, which is itself an interesting and challenging problem, we intend to discuss the functional meaning of the two circuits. To this end, we could assign a dynamics to the network and try to decipher the implications of the rewiring. Interestingly, we found that GI is recruited to save the network: if we leave GI as it is in the mock case, the whole network will rapidly converge to a fixed point state (a dead state). We will publish the results elsewhere.

### Reasons for short size data

In our synthesized data, we test both short and long data samples and come to the conclusion that there is a critical size, at which the two approaches behave differently. In our experimental data, we only tested it for the short data set. Of course, as we mentioned above, in neurophysiological data, we have recordings of long time traces and the Granger causality is widely used there. However, we have to realize that all *in vivo *recordings are very dynamic and stationary of data will become a key issue once we apply both approaches to a long dataset. Furthermore, when the dataset is long, both approaches could do well and it is more difficult to find the difference between the two. Hence we have only compared the results for short data length in the experimental setup.

### Reasons for small size of variables

In our synthesized data, we only used 5 variables to simulate a small interacting network; the number of variables could affect the result we derived. As expected, see also [23], the estimation of the Granger causality becomes unfeasible when the number of variables is large and the amount of the data sets is small. Hence, all results in the literature on estimating Granger causality are exclusive for small networks (around the order of 10), as we considered here. This is more or less true for dynamic Bayesian network inference as well. Extending the Granger causality and the dynamic Bayesian network inference to large networks is a challenging problem, even before we carry out the same comparison study on these two approaches as we did here.

## Conclusion

In this paper, we carried out a systematic and computationally intensive comparison between the two network structures derived from two common approaches: the dynamic Bayesian network inference and the Granger causality. These two approaches are applied on both synthesized and experimental data. For synthesized data (both linear model and non-linear model), a critical point of the data length is found, and the result is further confirmed in experimental data. The dynamic Bayesian network inference performs better than the Granger causality approach, when the data length is short, and vice versa.

## Methods

### Granger causality

Causal influence measurement notation for time series was firstly proposed by Wiener-Granger. We can determine a causal influence of one time series on another, if the predication of one time series can be improved by incorporating the knowledge of the second one. Granger applied this notation by using the context of linear vector auto-regression (VAR) model of stochastic processes [30-33]. In the AR model, the variance of the prediction error is used to test the perdition improvement. For instance, assume two time series; if the variance of the autoregressive prediction error of the first time series at the present time is reduced by inclusion of past measurements from the second time series, then one can conclude that the second time series have a causal influence on the first one. Geweke [15,16] decomposed the VAR process into the frequency domain, it converted the causality measurement into a spectral representation and made the interpretation more appealing.

The pairwise analysis introduced above can only be applied to bivairate time series. For more than two time series, a time series can have a direct or indirect causal influence to other time series. In this case, pairwise analysis is not sufficient or misleading for revealing whether the causal interaction between a pair is direct or indirect. In order to distinguish the direct and indirect causal affect, one introduces the conditional causality which takes account of the other time series' effect in a multivariate time series. In this paper, we used conditional causality to compare with the Bayesian network inference introduced before.

### Linear conditional Granger causality

The conditional Granger causality was defined by Granger. It can be explained as following. Giving Two time series **X**_*t *_and **Z**_*t*_, the joint autoregressive representation for **X**_*t *_and **Z**_*t *_by using the knowledge of their past measurement can be expressed as

(4)

and the noise covariance matrix for the system can be represented as

(5)

where var and cov represent variance and co-variance respectively. Incorporating the knowledge of third time series, the vector autoregressive mode can be represented involving three time series **X**_*t*_, **Y**_*t *_and **Z**_*t *_can be represented as

(6)

And the noise covariance matrix for the above system is

(7)

where **ε**_*it*_, *i *= 1, 2, ⋯, 5 are the prediction error, which are uncorrelated over time. From above two sets of equations, the conditional Granger causality form **Y **to **X **conditional on **Z **can be defined as

(8)

When the causal influence from **Y **to **X **is entirely mediated by **Z**, the coefficient *b*_2*i *_is uniformly zero, and the two autoregression models for two time series and three time series will be exactly same, thus we can get var(**ε**_1*t*_) = var(**ε**_3*t*_). We then can deduce *F*_**Y→X|Z **_= 0, which means **Y **can not futher improve the prediction of **X **including past measurements of **Y **conditional on **Z**. For var(**ε**_1*t*_) > var(**ε**_3*t*_) and *F*_**Y→X|Z **_= 0, we can say that there is still a direct influence from **Y **to **X **conditional on the past measurements of **Z**.

### Non-linear conditional Granger causality

We can extend our Granger causality to a non-linear model by using a series kernel functions [22,34]. Let **X**, **Y **and **Z **be three time series of n simultaneously measured quantities, which are assumed to be stationary. We are supposed to quantify how much **Y **cause **X **conditional on **Z**. The general expression for the nonlinear model is:

(9)

Function Φ can be selected as the kernel function of **X **and **Z **which has the following expression:

(10)

(11)

where ,  are centers of **X **and **Z**, ,  are variances of **X **and **Z**. The covariance matrix of prediction error can be expressed as

(12)

A joint autoregressive representation has the following expression:

(13)

The covariance matrix of prediction error can be expressed as

(14)

Similarly, we can define the conditional causality as

(15)

### Bayesian network

Bayesian networks are probabilistic graphical models initially introduced by [Kim & Pearl, 1987]. A Bayesian network is the specific type of graphical model which is directed acyclic graph [35,36]. Each arc in the model is directed and there is no way to start from any nodes and travel along a set of directed edges and get back at the initial node. The set of nodes represent a set of random variables [**X**_1_, **X**_2_, ⋯, **X**_*n*_], and the arcs express statistical dependence between the downstream variables and the upstream variables. The upstream variables are also called the parent variables of the downstream variables. Bayesian network inference yields the most concise model, automatically excluding arcs based on dependencies already explained by the model, which means the arcs in the network can be interpreted as a conditional causality. The edges in the Bayesian network encode a particular factorization of the joint distribution. The joint probability distribution can be decomposed as following:

(16)

That is, the joint probability distribution is the product of the local distributions of each node and its parents. If node **X**_*i *_has no parents, its local probability distribution is said to be unconditional, otherwise it is conditional. This decomposition is useful for Bayesian networks inference algorithm to deal with the uncertain situation and incomplete data.

To learn the parameter of the Bayesian network is to essentially estimate two kinds of probability distributions: the probability P(**X**) and the conditional probability P(**X**|**Y**). There are two kinds of approaches to density estimation; the nonparametric method and the parametric method. The easiest estimation for nonparametric method is to use the histogram approach. The distribution can then be a tabular conditional probability distribution, which is represented as a table. However this approach requires a much larger sample size to give us an accurate estimation, which is not suitable for general experimental data. For parametric method, one needs to make some assumptions about the form of the distribution such as widely used Gaussian distribution. For a D-dimensional vector **X**, the multivariate Gaussian distribution is in the form

(17)

Where **μ **is a D-dimensional mean vector, **Σ **is a **D **× **D **covariance matrix, and |**Σ**| denotes the determinant of **Σ**. In this paper, we first consider every node's conditional probability distribution as a conditional Gaussian distribution for the following inferences. The distribution on a node **X **can be defined as follows:

(18)

(19)

Where the **T **is the matrix transposition. **W **is the connection weight vector between node **X **and its parents **Y**. It can be represented by using the covariance matrix as following:

(20)

The detailed inductions of parameter estimations are given in the next chapter.

For learning the structure of the Bayesian network, one needs to search the space of all the possible structures and find out the best one which can be used to describe the input data, which is to maximum the conditional probability P(*Data*|*θ, M*) of data (*Data*) by give the parameters (*θ*) and the network structure (*M*). In order to balance the complex and concise of the structure, we can use BIC (Bayesian Information Criterion) as a scoring function, which includes an extra penalty term.

### Parameter learning for linear Gaussian

The parameter learning part can be approached by fitting a linear-Gaussian model. The goal of learning parameter in Bayesian network is to estimate the mean and covariance of the conditional Gaussian distribution, thus we can deduce the parameters of **μ, σ **and **W **in the equation (19).

Suppose **X **is a D-dimensional vector with Gaussian distribution N(**X**|**μ, Σ**), and one partition **X **into two disjoint subsets **X**_*a *_and **X**_*b*_, To be easily illustration, one takes the first M components of **X **to form **X**_*a*_, and the remaining components to form **X**_*b*_, so that

(21)

We also define mean vector **μ **and the covariance matrix **Σ **given by

(22)

Considering the quadratic form in the exponent of the Gaussian distribution, we can get following equation by a transformation.

(23)

From these and regard **X**_*b *_as a constant, we obtain the following expressions for the mean and covariance of the conditional probability distribution P(**X**_*a*_|**X**_*b*_).

(24)

(25)

Thus the parameters in the Bayesian network can be learned from above two equations.

### Parameter learning for non-linear Gaussian

We can also extend our linear model to a non-linear model like that for the Granger causality case. Suppose we have two variables which can be expressed as in equation (9). The kernel function is also chosen as described in equation (10) and equation (11).

Our non-linear model, the probability distribution for **X**_*t *_is no longer a Gaussian distribution. From the expression in equation (9), we can find that the probability distribution for **X**_*t *_is a combined distribution of kernel function distribution for the past measured values of **X **and **Z**, and a Gaussian distribution for the noise term. The kernel distribution is very difficult to derive, so one can use a mixture of Gaussian models to approximate the real distribution of kernel function. The mixture Gaussian model is in the form:

(26)

Each Gaussian density N(**X**|**μ**_*k*_, **Σ**_*k*_) is called a component of the mixture and has its own mean **μ**_*k *_and covariance **Σ**_*k*_. The parameter *π*_*k *_are called mixing coefficients which satisfies:

(27)

The conditional probability distribution for **X**_*t *_conditional on the past observation of **X **and **Y **in the nonlinear model is still a Gaussian distribution which can be easily obtained as following:

(28)

where *w *is the connection weights between node **X **and it parents. It can be estimated by using the simple linear regression method.

### Structure learning

There are two very different approaches to structure learning: one is constraint-based and the other is search and score algorithm. For the constraint-based algorithm, we start with a fully connected network and then remove the arcs, which are conditional independent. This has the disadvantage that repeated independence tests lose statistical power. For the latter algorithm, we perform a search on all possible graphs and select one graph which best describes the statistical dependence relationship in the observed data.

Unfortunately, the number of possible graphs increases super-exponentially with the number of nodes, so some search algorithms are required for overcoming this kind of complex problem rather than doing a exhaustive search in the space. There are several searching algorithms that can be applied; such as annealing search, genetic algorithm search and so on. The question could become easier if we know the total order of the nodes. The K2 algorithm allows us to find the best structure by selecting the best set of parents for each node independently. In the dynamic Bayesian networks, the order of nodes can be interpreted as the sequence of time lags represented for each node, so the K2 algorithm is applied for Bayesian network structure learning in this paper (see Appendix 2 in Additional file 1 for more details descriptions). The K2 algorithm tests parent insertion according to the order. The first node cannot have any parent, for other nodes, we can only choose the parent nodes which are behind it in this order. Then the scoring function can be applied to determine the best parent set, i.e. the one which gives the best score.

In addition to the search algorithm, a scoring function must be defined in order to decide which structure is the best (a high scoring network). There are two popular choices. One is the Bayesian score metric which is the marginal likelihood of the model, and the other is BIC (Bayesian Information Criterion) defined as following:

(29)

Where *Data *is the observed data, *θ *is the estimated value of the parameters, d is the number of parameters and N is the number of data cases. The term of  is regarded as a penalty term in order to balance both simple and accurate structure representation.

Suppose we observed a set of independent and identically distributed data *Data *= {**Y**^1^, **Y**^2^,..., **Y**^*N*^}, each of which can be a case of multi-dimensional data. Then the log likelihood of the data set can be defined as

(30)

Where j is the index of the nodes or variables in the Bayesian network, *pa*(*j*) is the set of parents of node j, and *θ*_*j *_are the parameters that define the conditional probability of *Y*_*j *_giving its parents.

In this paper, the Bayesian networks inference can then be approached by following procedure: initially, K2 algorithm is applied to search the space of possible graphs. For each possible structure, we can use the parameter learning algorithm to estimate the parameters of the networks. The BIC scoring function assigns a corresponding score through the estimated parameters and observed data set. The best network we can get is the highest score structures among all the possible graphs [37].

## Authors' contributions

The whole work was carried out by CZ and supervised by JF.

## Supplementary Material

Additional file 1 **A method for Bayesian network inference approach in a frequency domain and a detailed description of Bayesian network structure learning.** Click here for file

Additional file 2 **A matlab software for Bayesian network inference approach used in this paper.** Click here for file
