# Granger causality vs. dynamic Bayesian network inference: a comparative study

**DOI:** 10.1186/1471-2105-10-401

**Published:** 2009-12-07

**Authors:** Cunlu Zou, Katherine J Denby, Jianfeng Feng

**Affiliations:** 1 Department of Computer Science, University of Warwick, Coventry, UK; 2 Warwick HRI and Warwick Systems Biology Centre, Warwick, UK; 3 Centre for Computational Systems Biology Fudan University, Shanghai, PR China

## Correction

After publication of this work [1], we noted that we inadvertently failed to include the complete list of all coauthors. The full list of authors has now been added and the Authors' contributions and Acknowledgements section modified accordingly.

## Authors' contributions

The whole work was carried out by CZ and supervised by JF. The experimental data was provided by KD.
