# Supplementary material for: Granger causality vs. dynamic Bayesian network inference: a comparative study
Source: BMC Bioinformatics. 2009 Apr 24;10:122. doi: 10.1186/1471-2105-10-122 (PMC2691740; doi:10.1186/1471-2105-10-122)
Supplement: Additional file 1 — A method for Bayesian network inference approach in a frequency domain and a detailed description of Bayesian network structure learning. [file 1471-2105-10-122-S1.doc]

**Appendix 1.**

To extend our Bayesian network inference approach to a frequency domain, one can apply a similar spectral decomposition procedure like that for the Granger causality case. Suppose we have three variables $\mathbf{X}_{t}$, $\mathbf{Y}_{t}$ and $\mathbf{Z}_{t}$, then we can transfer our learned Bayesian Network parameters and structure to the polynomial equations. First, the joint autoregressive representation of $\mathbf{X}_{t}$ and $\mathbf{Z}_{t}$ can be represented as equation (4), where $a_{1i}$,$b_{1i}$,$c_{1i}$ and $d_{1i}$ are the connection weights between two nodes in the dynamic Bayesian network. These connection weights can be estimated by using equation (20) in the parameter learning method chapter. If there is no arc between two nodes, then the corresponding connection weight equals zero. The covariance matrix of the noise term is shown in equation (5). This covariance matrix can also be estimated in our parameter learning method by using equation (25). Next we consider the joint autoregressive representation by adding the variable $\mathbf{Y}_{t}$ into the system shown in equation (6), where the covariance matrix of the noise term is represented in equation (7). To derive the spectral decomposition of the time domain Bayesian network, we multiply the normalization matrix

$$P_{1}=\left(\begin{matrix} I & 0 \\ -\mathbf{S}_{21}\mathbf{S}_{11}^{-1} & I \end{matrix}\right)$$

to the both side of equation (4) and rewrite it in terms of the lag operator. where $I$ is identity matrix.

$$\left(\begin{matrix} D_{11}(L) & D_{12}(L) \\ D_{21}(L) & D_{22}(L) \end{matrix}\right)\left(\begin{matrix} \mathbf{X}_{t} \\ \mathbf{Z}_{t} \end{matrix}\right)=\left(\begin{matrix} \mathbf{X}_{t}^{*} \\ \mathbf{Z}_{t}^{*} \end{matrix}\right)$$

Then we can apply a same normalization procedure to the equation (6) by multiplying the matrix

$$P=P_{3}\cdot P_{2}$$

where

$$P_{2}=\left(\begin{matrix} I & 0 & 0 \\ -\mathbf{\Sigma}_{\mathbf{yx}}\mathbf{\Sigma}_{\mathbf{xx}}^{-1} & I & 0 \\ -\mathbf{\Sigma}_{\mathbf{zx}}\mathbf{\Sigma}_{\mathbf{xx}}^{-1} & 0 & I \end{matrix}\right)$$

and

$$P_{3}=\left(\begin{matrix} I & 0 & 0 \\ 0 & I & 0 \\ 0 & -(\mathbf{\Sigma}_{\mathbf{zy}}-\mathbf{\Sigma}_{\mathbf{zx}}\mathbf{\Sigma}_{\mathbf{xx}}^{-1}\mathbf{\Sigma}_{\mathbf{xy}})(\mathbf{\Sigma}_{\mathbf{yy}}-\mathbf{\Sigma}_{\mathbf{yx}}\mathbf{\Sigma}_{\mathbf{xx}}^{-1}\mathbf{\Sigma}_{\mathbf{xy}})^{-1} & I \end{matrix}\right)$$

to both sides of equation (6) and rewrite it in terms of the lag operator.

$$\left(\begin{matrix} B_{11}(L) & B_{12}(L) & B_{13}(L) \\ B_{21}(L) & B_{22}(L) & B_{23}(L) \\ B_{31}(L) & B_{32}(L) & B_{33}(L) \end{matrix}\right)\left(\begin{matrix} \mathbf{X}_{t} \\ \mathbf{Y}_{t} \\ \mathbf{Z}_{t} \end{matrix}\right)=\left(\begin{matrix} \mathbf{\varepsilon}_{xt} \\ \mathbf{\varepsilon}_{yt} \\ \mathbf{\varepsilon}_{zt} \end{matrix}\right)$$

After Fourier transforming equation (32) and (36), we can rewrite them in the following representations.

$$\left(\begin{matrix} \mathbf{X}(\lambda) \\ \mathbf{Z}(\lambda) \end{matrix}\right)=\left(\begin{matrix} G_{\mathbf{xx}}(\lambda) & G_{\mathbf{xz}}(\lambda) \\ G_{\mathbf{zx}}(\lambda) & G_{\mathbf{zz}}(\lambda) \end{matrix}\right)\left(\begin{matrix} \mathbf{X}^{*}(\lambda) \\ \mathbf{Z}^{*}(\lambda) \end{matrix}\right)$$

$$\left(\begin{matrix} \mathbf{X}(\lambda) \\ \mathbf{Y}(\lambda) \\ \mathbf{Z}(\lambda) \end{matrix}\right)=\left(\begin{matrix} H_{\mathbf{xx}}(\lambda) & H_{\mathbf{xy}}(\lambda) & H_{\mathbf{xz}}(\lambda) \\ H_{\mathbf{yx}}(\lambda) & H_{\mathbf{yy}}(\lambda) & H_{\mathbf{yz}}(\lambda) \\ H_{\mathbf{zx}}(\lambda) & H_{\mathbf{zy}}(\lambda) & H_{\mathbf{zz}}(\lambda) \end{matrix}\right)\left(\begin{matrix} \mathbf{E}_{\mathbf{x}}(\lambda) \\ \mathbf{E}_{\mathbf{y}}(\lambda) \\ \mathbf{E}_{\mathbf{z}}(\lambda) \end{matrix}\right)$$

Assuming that $\mathbf{X}(\lambda)$ and $\mathbf{Z}(\lambda)$ from equation (37) can be equated with the equation (38), we combine both equations to yield.

$$\left(\begin{matrix} \mathbf{X}^{*}(\lambda) \\ \mathbf{Y}(\lambda) \\ \mathbf{Z}^{*}(\lambda) \end{matrix}\right)=\left(\begin{matrix} G_{\mathbf{xx}}(\lambda) & 0 & G_{\mathbf{xz}}(\lambda) \\ 0 & I & 0 \\ G_{\mathbf{zx}}(\lambda) & 0 & G_{\mathbf{z}z}(\lambda) \end{matrix}\right)^{-1}\left(\begin{matrix} H_{\mathbf{xx}}(\lambda) & H_{\mathbf{xy}}(\lambda) & H_{\mathbf{xz}}(\lambda) \\ H_{\mathbf{yx}}(\lambda) & H_{\mathbf{yy}}(\lambda) & H_{\mathbf{yz}}(\lambda) \\ H_{\mathbf{zx}}(\lambda) & H_{\mathbf{zy}}(\lambda) & H_{\mathbf{zz}}(\lambda) \end{matrix}\right)\left(\begin{matrix} \mathbf{E}_{\mathbf{x}}(\lambda) \\ \mathbf{E}_{\mathbf{y}}(\lambda) \\ \mathbf{E}_{\mathbf{z}}(\lambda) \end{matrix}\right)$$

$$=\left(\begin{matrix} Q_{\mathbf{xx}}(\lambda) & Q_{\mathbf{xy}}(\lambda) & Q_{\mathbf{xz}}(\lambda) \\ Q_{\mathbf{yx}}(\lambda) & Q_{\mathbf{yy}}(\lambda) & Q_{\mathbf{yz}}(\lambda) \\ Q_{\mathbf{zx}}(\lambda) & Q_{\mathbf{zy}}(\lambda) & Q_{\mathbf{zz}}(\lambda) \end{matrix}\right)\left(\begin{matrix} \mathbf{E}_{\mathbf{x}}(\lambda) \\ \mathbf{E}_{\mathbf{y}}(\lambda) \\ \mathbf{E}_{\mathbf{z}}(\lambda) \end{matrix}\right)$$

Where $Q(\lambda)=G^{-1}(\lambda)H(\lambda)$. The power spectrum of $\mathbf{X}^{*}(\lambda)$ is found to be

$$S_{\mathbf{x}^{*}\mathbf{x}^{*}}=Q_{\mathbf{xx}}(\lambda)\hat{\Sigma}_{\mathbf{xx}}Q'_{\mathbf{xx}}(\lambda)+Q_{\mathbf{xy}}(\lambda)\hat{\Sigma}_{\mathbf{yy}}Q'_{\mathbf{xy}}(\lambda)+Q_{\mathbf{xz}}(\lambda)\hat{\Sigma}_{\mathbf{zz}}Q'_{\mathbf{xz}}(\lambda)$$

The first term can be thougth of as the intrinsic power and the remaining two terms as the combined causal influences from $\mathbf{Y}$ on the mediate of $\mathbf{Z}$. This interpretation leads to the definition.

**Appendix 2.**

Initially, each variable can be interpreted as a sequence of nodes which represents as the different time lags. Suppose we observed a set of independent and identically distributed time series data $\mathbf{Y}=[Y^{1},Y^{2},\cdots,Y^{N}]$. Every node can represent a specific time lags for a specific variable, For instance, variable$j$ ($j\in[1,2,\cdots,N]$) can be interpreted as a sequence of nodes $[\cdots,Y_{t-3}^{j},Y_{t-2}^{j},Y_{t-1}^{j},Y_{t}^{j}]$. Hence, the total order of the nodes can then be $[\cdots,\mathbf{Y}_{t-3},\mathbf{Y}_{t-2},\mathbf{Y}_{t-1},\mathbf{Y}_{t}]$. Since one only concern the causal relation between different time lags, the order of various variables for the same time lags can be random selected. Then the potential parent set for every node can then be determined according to the total order, which contains all the nodes before it. Finally, we can apply the K2 algorithm to select the set of best parents from the set of potential parents for every node independently. This procedure is described as following pseudo code for a specific node .

| 1. Calculate **initialScore**: the initial BIC score for node $Y_{t}^{j}$ (initially no parents). 2. Test each node to be the parent node of $Y_{t}^{j}$ in the set of potential parents **S**, for each node, calculate the BIC score. 3. Select the best parent node (**bestNode**) which gives the highest score: **newScore**. 4. compare **newScore** to the **initialScore**, if smaller then stop, else add a arc from **bestNode** to the node . 5. change **initialScore** to **newScore**. 6. remove **bestNode** from set **S.** 7. go back to step 2. |
| --- |
